# TGF-β Signaling in Gastrointestinal Cancers: Progress in Basic and Clinical Research

**DOI:** 10.3390/jcm6010011

**Published:** 2017-01-18

**Authors:** Takehiko Yokobori, Masahiko Nishiyama

**Affiliations:** 1 Research Program for Omics-based Medical Science, Division of Integrated Oncology Research, Gunma University Initiative for Advanced Research, 3-39-22 Showa-machi, Maebashi, Gunma 371-8511, Japan; m.nishiyama@gunma-u.ac.jp; 2 Department of Molecular Pharmacology and Oncology, Gunma University Graduate School of Medicine, 3-39-22 Showa-machi, Maebashi, Gunma 371-8511, Japan

**Keywords:** TGF-β signaling, TGFBI, gastrointestinal cancer, EMT, tumor promoter, tumor suppressor

## Abstract

Transforming growth factor (TGF)-β superfamily proteins have many important biological functions, including regulation of tissue differentiation, cell proliferation, and migration in both normal and cancer cells. Many studies have reported that TGF-β signaling is associated with disease progression and therapeutic resistance in several cancers. Similarly, TGF-β-induced protein (TGFBI)—a downstream component of the TGF-β signaling pathway—has been shown to promote and/or inhibit cancer. Here, we review the state of basic and clinical research on the roles of TGF-β and TGFBI in gastrointestinal cancers.

## 1. Introduction

Transforming growth factor (TGF)-β signaling regulates various cellular processes including proliferation, apoptosis, differentiation, cytokine secretion, extracellular matrix (ECM) modification, and tumor migration [1,2]. The TGF-β superfamily includes not only the TGF-β ligands TGF-β1, TGF-β2, and TGF-β3 but also other growth factors such as Nodal, Activin, Lefty, bone morphogenetic proteins (BMPs), and differentiation factors [3]. Upon ligand binding, TGF-β receptors phosphorylate Smad 2 and 3. On the other hand, BMP receptors phosphorylate Smad 1, 5, and 8 [2,4]. These phosphorylated substrates translocate to the nucleus to regulate the transcription of several target genes—including cancer-associated genes such as snail, slug, and zinc finger E-box-binding homeobox (ZEB)1/2—which are known to promote epithelial-mesenchymal transition (EMT), metastasis, cancer stem cell maintenance, and angiogenesis [5,6].

The TGF-β pathway has attracted attention as both a cancer marker and therapeutic target in many diseases, including gastrointestinal cancers [6,7]. However, TGF-β signaling plays a dual role as a tumor suppressor in pre-malignant states and as a tumor promoter in advanced cancers [2]. Therefore, there is a need for therapeutic strategies that target the TGF-β signaling pathway, including cancer-related downstream genes without blocking the tumor-suppressor effects.

Recent advances in our understanding of TGF-β signaling have provided insight into the molecular basis of the relationship between TGF-β and cancer. In this review, we examine the evidence for TGF-β-induced protein (TGFBI; also known as βig-H3) as a therapeutic target of TGF-β signaling in gastrointestinal cancers. TGFBI is a downstream component of the TGF-β signaling pathway that has been implicated in corneal disorder, diabetes, and atherosclerosis as well as in the development and progression of several cancers. We also discuss the association between TGF-β and TGFBI and their significance in gastrointestinal cancers.

## 2. Role of TGF-β in Cancer

TGF-β acts as a tumor suppressor in premalignant tumor development and as a tumor promoter in advanced tumors, specifically during invasion and metastasis [8,9,10]. TGF-β signaling and downstream targets are reportedly downregulated in several malignancies including lung cancer, hepatocellular carcinoma, and breast cancer [11,12,13,14,15,16]. TGF-β signaling can induce EMT in cancer cells, which leads to metastasis [17]. EMT is an important cellular process that is associated with embryonic development, fibrosis, and tumorigenesis along with other diseases [18,19]. In addition to TGF-β, EMT is induced by Wnt, Notch, Hedgehog, receptor tyrosine kinases, hypoxia, and microRNAs [17]. TGF-β pathway activation regulates E- and N-cadherin, Snail, Slug, zinc finger E-box-binding homeobox (ZEB)1, and ZEB2 [2,18]. Inhibiting TGF-β signaling and thereby blocking EMT is an attractive strategy for preventing metastasis of advanced tumors.

### 2.1. Significance of TGF-β Expression in Gastrointestinal Cancers

The high expression of TGF-β was reported to be associated with cancer progression and metastasis in gastrointestinal cancers including esophageal cancer, gastric cancer, colon cancer, liver cancer, and pancreatic and biliary cancer [20,21,22,23,24,25]. Activation of TGF-β signaling promotes EMT induction and maintains the cancer stem cell properties [26,27,28]. Moreover, the expression of TGF-β in serum might be a useful cancer biomarker in gastrointestinal cancers [20,22,24]. TGF-β signaling is identified as both of a tumor suppressor and as a tumor promoter in cancers [2], however, the evaluation of TGF-β expression in tumor and serum is suggested to be a promising marker for poor prognosis and malignant potential in gastrointestinal cancers.

### 2.2. Role of TGFBI in Cancer

Among the downstream genes of TGF-β, TGFBI was focused as one of the candidates of EMT regulator because TGFBI expression was found to be upregulated in cholangiocarcinoma (CC) cells exhibiting mesenchymal sarcomatous changes relative to epithelial CC cells [29]. The mechanism by which TGFBI induces EMT is unclear; however, TGFBI is positively or negatively associated with cancer cell proliferation and invasion depending on the cancer type [30]. Further research is needed to clarify whether TGFBI modulates EMT in gastrointestinal cancers.

**Figure 1:**
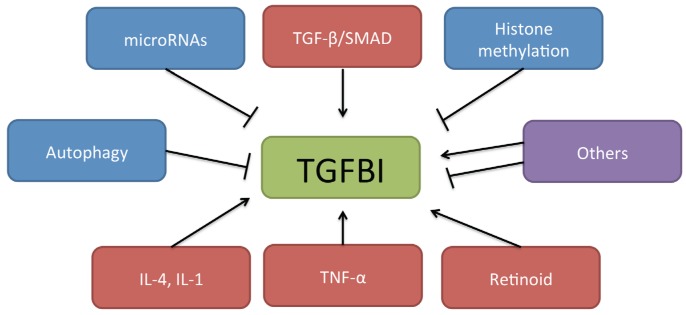
Mechanism of TGF-β-induced protein (TGFBI) regulation. TGFBI is regulated not only by TGF-β/SMAD signaling but also by other mechanisms including microRNAs, histone methylation, autophagy, interleukins, tumor necrosis factor-α, and retinoid.

TGFBI is a 68-kDa ECM protein that was first isolated from the A549 human lung adenocarcinoma cell line treated with TGF-β [31]. It contains 683 amino acids that include a secretory signal peptide sequence and a cysteine-rich EMI domain as well as four fasciclin 1 motifs and an arginine–glycine–aspartate motif. TGFBI is expressed in several organs including the skin, heart, liver, and pancreas [31], and has been linked to various diseases including corneal disorder [32], diabetes [33], nephropathy [34], wound healing [35], atherosclerosis [36], and many types of cancer. In esophageal cancer, secreted-TGFBI has been detected in the ECM and tumor vasculature by immunohistochemistry [37]. TGFBI expression is regulated not only by TGF-β, but also by other mechanisms including autophagy [38,39], microRNAs [40], retinoid [41], interleukin (IL)-1β, tumor necrosis factor-α [42], IL-4 [43], secreted *protein* acidic and rich in cysteine [44], 4-phenylbutyric acid [45], and cullin 4A [46] (Figure 1). The mechanism of TGFBI regulation merits more detailed investigation as a potential therapeutic target.

### 2.3. TGFBI in Gastrointestinal Cancers

TGFBI knockout mice show increased spontaneous tumor growth and susceptibility to chemically induced skin tumors as compared to wild-type mice [47], suggesting a tumor-suppressor function. On the other hand, TGFBI has also been shown to act as a tumor promoter in the gastrointestinal tract [48], and its overexpression in mice increased the rate of formation of spontaneous gastrointestinal and *N*,*N*-diethylnitrosamine-induced liver tumors. However, the rate of tumorigenesis is similar between TGFBI knockout and wild-type mice. As in the case of therapeutic approaches that target TGF-β in cancers, TGFBI inhibition may stimulate or suppress tumor growth, although several reports indicate that TGFBI promotes the growth of gastrointestinal tumors (Table 1).

#### 2.3.1. Esophageal Cancer

TGFBI expression was found to be upregulated in esophageal squamous cell carcinoma (ESCC) as compared to non-cancerous tissues, by microarray and reverse transcription PCR analyses. However, the significance of this observation is unclear. An examination of the relationship between TGFBI expression, clinicopathological findings, and patient prognosis revealed that TGFBI was mainly expressed in the stroma in ESCC, and patients with high stromal TGFBI expression had more frequent hematogenous recurrence and worse prognosis than those with low expression. This suggests that high levels of TGFBI in the stroma and not in tumor cells underlies tumor aggressiveness in ESCC.

#### 2.3.2. Gastric Cancer

TGFBI overexpression in the ECM induced the formation of gastric tumors [48], suggesting that TGFBI has an oncogenic function in gastric cancer. Moreover, bone marrow-derived mesenchymal stem cells were shown to induce proliferation, cluster formation, and expression of the cancer stem cell marker cluster of differentiation 133 and TGFBI in co-cultured MKN7 gastric cancer cells [49]. Moreover, serum levels of secreted TGFBI were higher in gastric cancer as compared to non-cancerous patients [48]. These observations suggest that soluble TGFBI in ECM stimulates carcinogenesis and an aggressive phenotype in surrounding gastric mucosa and cancer cells.

#### 2.3.3. Pancreatic and Biliary Cancer

Similar to its levels in patients with gastric cancer, serum TGFBI levels were higher in biliary carcinoma as compared to non-cancer patients [48]. Serum TGFBI concentration is therefore considered a potential biomarker in some gastrointestinal cancers; indeed, a proteomic analysis showed that TGFBI expression was higher in pancreatic ductal adenocarcinoma than in non-cancerous tissues [50].

TGFBI is downregulated in pancreatic islet cells of type 1 diabetes, and inhibits T cell activation and the production of cytotoxic molecules such as granzyme B and interferon-γ by blocking the autoimmune response via suppression of Lck tyrosine kinase [51]. TGFBI was found to be expressed in pancreatic cancer stem cells [52] and is thought to mediate immune tolerance through inhibition of cytotoxic T cell activation in pancreatic cancer.

TGFBI is overexpressed in non-*Opisthorchis viverrini*-related intrahepatic cholangiocarcinoma (CC) [53]; this is associated with sarcomatous changes such as EMT induction [29], which leads to aggressive intrahepatic spreading and metastasis in CC. As such, measurement of serum TGFBI levels may be useful for the diagnosis of EMT-induced CC with high potential for malignancy.

#### 2.3.4. Colorectal Cancer

TGFBI was found to be upregulated in colorectal cancer relative to corresponding adenomas and non-cancerous tissues [54]. Expression was mainly detected in the central stroma of colon cancer that had metastasized to the liver [55]. A functional analysis of TGFBI in various colon cancer cell lines revealed that high levels of the protein were associated with enhanced metastasis and extravasation [56]. These findings suggest that TGFBI expression in colon cancer is a marker of liver metastasis.

## 3. TGF-β and TGFBI as Molecular Targets

Clinical trials of drugs targeting the TGF-β pathway in several diseases [57,58,59,60,61,62] have shown that directly inhibiting TGF-β signaling can be an effective therapeutic strategy against refractory cancers, but may have severe side effects including the development of cutaneous malignancies. For instance, GC1008—an anti-TGF-β monoclonal antibody—induced the formation of cutaneous tumors including keratoacanthoma, squamous-cell carcinomas, and hyperkeratosis [62]. Future therapeutic approaches could target the downstream components of the TGF-β signaling pathway to block their cancer-promoting effects without inhibiting tumor suppression.

Overexpression of stromal TGFBI in a mouse model induced tumorigenesis of gastric mucosa and liver, but not in TGFBI knockout mice [48]. Moreover, serum TGFBI concentrations were higher in patients with cholangiocarcinoma, hepatocarcinoma, and gastric cancer than in non-tumor patients. These data suggest that TGFBI levels in the stroma, ECM, and serum may directly or indirectly promote gastrointestinal cancer development.

ECM expression of TGFBI was shown to be a useful marker for the response to paclitaxel in ovarian cancer [63]. Consistent with this observation, TGFBI was found to be a chemo-sensitizer to paclitaxel, cisplatin, and gemcitabine in lung cancer cell lines, while upregulation of TGFBI expression was associated with response to chemotherapy in lung cancer patients. Moreover, recombinant TGFBI induced apoptosis in cancer cells via integrin activation [64]. In melanoma, mesothelioma, and breast cancer, TGFBI overexpression suppressed proliferation and malignant potential [65,66,67]; however, targeting TGFBI may be associated with an increased risk of chemo-resistance in these patients. Further research should be performed to clarify whether TGFBI can function as a chemo-sensitizer or risk of chemo-resistance in gastrointestinal cancers.

## 4. Concluding Remarks

Dysregulation of TGFBI has been observed not only in cancers but also in corneal dystrophy and diabetes. Like TGF-β, TGFBI acts as both a tumor suppressor and promoter in several types of cancer and may be a useful therapeutic target in gastrointestinal tumors. However, the relationship between TGFBI expression and the development of chemosensitivity must be clarified since targeting TGFBI may abrogate the cytotoxic effects of chemotherapy. Combining a TGFBI inhibitor with other anticancer drugs may be an effective treatment strategy for gastrointestinal cancers that can preserve the tumor-suppressor effect of TGF-β signaling and TGFBI.

## Figures and Tables

**Table 1:** Significance of TGFBI expression in gastrointestinal tumors.

Tumor Type	Significance	PMID Reference
Esophageal tumor	TGFBI expression was higher in the extracellular matrix (ECM) of tumors as compared to normal tissues.	19082484
	High TGFBI was associated with tumor progression and poor prognosis in esophageal cancer.	25448803
Gastric tumor	TGFBI overexpression in stomach tissue of TGFBI knock-in mice caused gastric tumors. Serum TGFBI level was higher in gastric cancer than in non-cancer patients.	25889002
	Bone-marrow-derived mesenchymal stem cells induced TGFBI expression in co-cultured gastric cancer cells.	22688186
Pancreatic and biliary tumor	Serum TGFBI expression was higher in pancreatic and biliary carcinoma patients than in non-cancer patients.	25889002
	TGFBI expression in pancreas inhibited T-cell activation and production of cytotoxic molecules including granzyme B and interferon-γ via suppression of Lck tyrosine kinase.	26470788
	TGFBI was overexpressed in pancreatic cancer stem cells.	23679566
	Proteomic analysis revealed that TGFBI was upregulated in pancreatic cancer as compared to non-cancer tissues.	21755970
	TGF-β induced *TGFBI* mRNA expression in pancreatic cancer cells as compared to normal control tissue.	12379307
	TGFBI was overexpressed in non-*Opisthorchis viverrini*-related intrahepatic cholangiocarcinoma.	17006947
	TGFBI upregulation was associated with sarcomatous changes such as EMT induction in cholangiocarcinoma.	19287191
Colorectal tumor	*TGFBI* mRNA level was higher in colon cancer than in adenomas and non-cancerous tissue.	11585723
	TGFBI secretion in colon cancer cells was found to be related to cancer aggressiveness and extravasation.	18245446
	Stromal TGFBI expression was higher in metastatic as compared to normal liver tissue. TGFBI expression was localized in the center part of the metastatic area.	23832580
